# 1724. Varicella Infections and Vaccination Rates Among Pediatric Solid Organ Transplant Recipients: A Single-Center, Multi-Organ Retrospective Experience (2011-2021)

**DOI:** 10.1093/ofid/ofad500.1556

**Published:** 2023-11-27

**Authors:** Gabriela Espinoza-Candelaria, Michael D Green, Judith M Martin, Jorna Sojati, Marian G Michaels

**Affiliations:** UPMC Children's Hospital of Pittsburgh, Pittsburgh, Pennsylvania; University of Pittsburgh School of Medicine, Pittsburgh, PA; University of Pittsburgh, Pittsburgh, Pennsylvania; University of Pittsburgh School of Medicine, Pittsburgh, PA; UPMC Children's Hospital of Pittsburgh, Pittsburgh, Pennsylvania

## Abstract

**Background:**

The two-dose varicella-zoster virus (VZV) immunization schedule significantly decreased VZV infections in the US. Yet, among pediatric solid organ transplant recipients (SOT), the characterization of pre-transplant VZV immunity, VZV exposure with progression to disease, and disease outcomes remain poorly described.

**Methods:**

We performed a retrospective analysis of patients < 18 years old who received a SOT between Jan 1, 2011 - Dec 31, 2021, at the UPMC Children's Hospital of Pittsburgh, a quaternary, multi-organ pediatric care center. Inpatient, outpatient, and telephone encounters were reviewed as documented in the patients’ electronic health records (EHRs) via automated and manual data extraction.

**Results:**

674 patients underwent 832 SOT procedures; 370 children met the inclusion criteria. 287 patients had documentation of their VZV pre-transplant vaccine status and no contraindication for vaccination; 59% had unknown, negative, or indeterminate pre-transplant VZV IgG. While 257 children received at least 1 VZV dose before SOT, another dose was administered for only 41% of those with negative or indeterminate serology in the pre-transplant period. 35 children did not have a documented VZV pre-transplant immunization status; 63% had unknown or negative VZV IgG and were eligible for vaccination by age and immunocompetence. 33 patients had VZV exposures; 2 children developed VZV disease - neither had received post-exposure prophylaxis (PEP). 5 patients were prescribed an antiviral as PEP, 2 received IVIG incidentally, and none received VZV immunoglobulin. In total, there were 4 primary VZV and 4 herpes zoster (HZ) medically attended cases among 8 patients. All 8 received antiviral treatment; 3 in the inpatient setting and 5 exclusively outpatient. All cases were limited to cutaneous lesions without dissemination. None of the patients who developed primary VZV had conclusive evidence of protective VZV IgG pre-transplant. Post-herpetic neuralgia complicated 1 HZ case.

**Conclusion:**

VZV exposure and disease still occur, albeit at low levels and with mild manifestations, among pediatric SOT recipients in the two-dose VZV vaccine era. Optimization of VZV immunity in the pre-transplant period and preventing progression to disease after exposure to VZV remain goals of care.

**Disclosures:**

**Michael D. Green, MD, MPH**, ADMA: Advisor/Consultant|Allovir: Advisor/Consultant|Bristol Myers Squibb: Advisor/Consultant|ITB-MED: Advisor/Consultant **Judith M. Martin, MD**, Merck, Sharp and Dhome: Grant/Research Support **Marian G. Michaels, MD, MPH**, Merck: Grant/Research Support|Viracor: Grant/Research Support

